# Assessment of particulate matter exposure levels and associated respiratory symptoms among oil seed processing factories workers

**DOI:** 10.1038/s41598-026-50211-z

**Published:** 2026-05-04

**Authors:** Bizuneh Ayano, Seblework Mekonen, Dessalegn Dadi

**Affiliations:** 1 Department of Environmental Health Science and Technology, Jimma University, Jimma, Ethiopia; 2 College of Medicine and Health Science, Department of Public Health, Salale University, Fiche, Ethiopia; 3 Institute of Water, Environment and Climate Research, Center for Water Research, Water and Public Health, Addis Ababa University, Addis Ababa, Ethiopia

**Keywords:** Particulate matter, Concentration, Respiratory symptoms, Factors, Oil seeds, Factories, Diseases, Environmental sciences, Health care, Medical research, Risk factors

## Abstract

Studies have shown that levels of fungal contamination and mycotoxin concentrations in oil-producing seeds vary across regions. In addition, extreme exposure to seeds dust particulate matter can lead to respiratory health problems. In Ethiopia oil-seeds processing factories workers exposure to particulate matter and magnitude of its respiratory symptoms have not been widely explored. To determine particulate matter concentration exposure levels and identify factors associated with respiratory symptoms among oil-seed processing factories workers. Using cross-sectional study design, 14 factories and 716 participants were selected through simple random sampling. Qualitative data were collected through checklist-based observation, in-depth interviews & FGDs and analyzed using qualitative content analysis. Direct particulate matter concentrations were measured using PATS + , Dylos DC1900, and Laser PM2.5 Meter-5800D/5800E devices. Descriptive statistics were used for data summarization, and analysis was conducted using STATA version 16.1. Binary logistic regression &multivariable logistic regression analysis were performed to identify associated factors, with p < 0.05 considered statistically significant. Among 716 participants, 49.9% were female. The mean PM₂.₅ and PM₁₀ concentrations in oil-seeds processing factories were 19.72 ± 11.8 µg/m^3^ and 55.24 ± 14.5 µg/m^3^, respectively. Respiratory symptoms and bronchitis were reported by 25.28% of workers. Wheezing (23.2%), phlegm (22.5%), dyspnea (22.3%), and occupational asthma (12.57%) were the most common symptoms. Significant predictors included particulate matter exposure [AOR = 18.6, 95% CI: (9.12, 37.43)], not PPE use [AOR = 18.3, 95% CI: (2.39, 39.39)], use of wood as an energy source [AOR = 15.47, 95% CI: (4.25,36.4)], and inadequate ventilation [AOR = 14.29,95% CI:(3.01–26.70)]. Workplace exposure to oil seeds PM_2.5_ and PM_10_ among oil-seeds processing factories workers exceeded WHO guideline limits. Respiratory and asthma symptoms were highly prevalent, with greater risk among workers exposed above WHO standards. Oil seeds particulate matter exposure, not personal protective Equipment use, energy source, insufficient ventilation, and training were significant factors associated with respiratory symptoms. Necessary measures are needed to limit particulate matter exposure, including effective occupational health and safety programs that emphasize source-level dust control, routine supervision, engineering controls (e.g., adequate ventilation), administrative actions (such as training and risk assessment), and appropriate personal protective equipment use. Future study of personal measurement with case control is desirable.

## Introduction

Particulate matter (PM) is a complex mixture of fine solid particles and fluid precipitations deferred in the atmosphere. These subdivisions are classified based on their aerodynamic diameter and differ in size, configuration, and origin. Among them, PM₂.₅ particles with diameters of 2.5 µm or smaller is of particular concern due to its ability to penetrate deep into the respiratory system and even enter the bloodstream. PM₁₀ refers to subdivisions with diameter of 10 µm or less. These two categories are the most extensively studied forms of particulate matter^1^. According to various studies, common respiratory symptoms among textile workers include coughing, phlegm production, wheezing, shortness of breath, chest tightness, and chronic bronchitis^2^. Research conducted in China revealed that approximately 32% of textile mill workers were affected by byssinosis^3^.

Scientific evidence indicates that uncontrolled atmospheric contamination resulting from rapid industrialization, population growth, and urban expansion contributes significantly to the deterioration of air quality^4,5^. Previous studies classify dust into two main types: inorganic and organic. Inorganic dust originates from materials such as stone, chemicals, and metals, including cement, coal, and asbestos^4,5^. In contrast, organic dust arises from plant, animal, and microbial sources^6^. Several studies report that organic dust is frequently released into the environment during various industrial and agricultural activities, including grain, sugarcane, coffee, and cotton processing^7^. In oilseed processing (OSP), dust particles are generated at different stages of seed handling and processing and are categorized as organic dust because they originate from plant-based seeds.

Research indicates that dust in oilseed processing factories contains particles ranging in size from 5 µm to 400 µm^8,9^. Oilseed processing involves several mechanical operations, including cleaning debris from raw seeds, hulling, grading, hand-picking, and wrapping. Primary seed processing typically occurs in storage areas or is sometimes transferred to other facilities, including those in other countries. Secondary processing includes refining, roasting, and cleaning procedures, as described in previous studies^6–8^. Additionally, one study reported that before industrialization, agriculture, basic food processing, and extractive industries were common sources of particulate matter (PM) dust exposure^10^. These particles are commonly released into the environment from industrial and agricultural activities such as grain, sugarcane, coffee, and cotton processing, as well as from seed, swine, and dairy production facilities^11,12^. Research also specifies that organic dust frequently contains biological components such as bacteria, molds, endotoxins, and mycotoxins^12,13^.

The World Health Organization (WHO) reported non-communicable diseases accounted for 82% of global deaths, with chronic respiratory diseases, including asthma, responsible for about 4 million deaths, or 10.7% of the total as stated by studies^14^. Study explained that in the United Kingdom, roughly 12,000 deaths happen annually due to occupational respiratory illnesses, about two-thirds of which are attributed to dust exposure^14^. According to World Health Organization (WHO), report more than one billion people worldwide suffer from chronic respiratory conditions, and approximately four million die prematurely each year from these diseases^15^. The Global Burden of disease study estimated that approximately 3.22 million deaths were attributable to exposure to air pollution, representing an increase from the previously reported 2.91 million deaths^16^. In addition, cancers of the trachea, bronchus, and lung account for nearly 7% of total mortality associated with exposure to PM_2.5_^16^.

Exposure to occupational airborne particulate matter is estimated to cause around 386,000 deaths and nearly 6.6 million disability-adjusted life years (DALYs) among employees, rendering to other studies^17^. Exposure to PM has been strongly linked to increased morbidity and mortality from respiratory, cardiovascular, and cerebrovascular diseases. Outdoor air pollution has been classified as a Group 1 carcinogen (carcinogenic to humans) by research on Cancer^18^. Furthermore, estimates from the World Health Organization indicate that air pollution was responsible for more than seven million premature deaths globally in a single year, with over 80% occurring in the Pacific and South Asian regions^18^.

Fine particulate matter, particularly PM_2.5_ (particles < 2.5 µm in aerodynamic diameter), is a major contributor to global mortality. Short-term increases in PM₂.₅ exposure are associated with a 1–3% rise in all-cause and cardiovascular mortality for every 10 μg/m^3^ increase^19^. Moreover, long-term exposure results in substantially greater health risks; for example, a large cohort study in Canada reported that a 10 μg/m^3^ increase in long-term PM₂.₅ exposure was associated with 15% and 31% higher risks of non-accidental and ischemic heart disease mortality, respectively^19^. Prolonged exposure may promote chronic cardiovascular conditions, including accelerated atherosclerosis^19^. Estimates indicate that particulate air pollution contributes to approximately 4.2 million premature deaths worldwide^20^. Projections further suggest that, by 2060, outdoor air pollution could result in 6–9 million premature deaths annually, with an associated economic burden equivalent to nearly 1% of global gross domestic product (approximately USD 2.6 trillion)^20^. Several studies have reported a strong positive association between exposure to particulate matter (PM) and mortality. Conversely, reductions in PM_2.5_ concentrations are associated with an increase in estimated mean life expectancy^21^.

Study claimed that chronic respiratory diseases (CRDs) in low-and middle-income countries interpretation for over 85% of all global CRD cases^22^. In African studies reported incidence of respiratory symptoms was relatively high, with rates of 46.3% in Zambia^23^, 43% in Namibia^24^, and 35.1% in Tanzania^25^.

In many processing factories in Ethiopia, studies have found that significant dust generation often results from the use of outdated machinery and technology^13^. Literatures have described that the highest total dust meditations 0.4, 5.8, 6.9, and 8.7 mg/m^3^ were detected in flour mills, grain scrubbing, padding, and unloading areas, correspondingly^26^. Though, some investigate has noted that respirable dust levels in certain flour mill workplaces can range as high as 160 mg/m^3^^2,27^. The highest points of dust experience were pragmatic in large-scale nutriment dispensation industries, with median concentration of 7.6 mg/m^3^, shadowed by medium-sized plants at 5.2 mg/m^3^ and small to micro-scale facilities ranging from 2.2 to 3 mg/m^3^^2^.

In recent years, studies have publicized that edible oils are increasingly being produced from plant-derived oil-seeds^28,29^. Numerous studies have documented fungal contamination in a wide range of oil-bearing seeds, as well as the occurrence of mycotoxins in the oils extracted from them^28,29^. Regulatory bodies such as the Food and Agriculture Organization (FAO), the Codex Alimentarius Commission, the European Union (EU) Commission, and the World Health Organization (WHO) have established strict regulations specifying the maximum permissible limits of mycotoxin contamination in oilseeds^29^. Studies have revealed that the level of fungal contamination and mycotoxin concentrations in oil-producing seeds and their derived oils vary by region^28,29^. Evidence from existing literature clearly designates that commercially produced oil-seeds and their edible oils are susceptible to contamination by toxigenic fungi and mycotoxins during both pre-harvest and post-harvest stages^28^. In particular, soybean and maize are highly vulnerable to fungal infestation and subsequent mycotoxin contamination under favorable environmental conditions, occurring both in the field and during storage or processing according to studies^28–30^. Commercially produced oilseeds and their edible oils are widely acknowledged to be susceptible to contamination by toxigenic fungi and mycotoxins during both pre-harvest and post-harvest periods^31^. This suggests that dust generated from oilseed processing factories may pose adverse respiratory risks to workers. Soybean, a widely cultivated oilseed, has been shown to contribute to the stabilization of contaminated environments through its Phyto stabilization potential for heavy metals, particularly copper (Cu)^32,33^. Additionally, literature indicates that metal accumulation in seeds can facilitate the transfer of heavy metals to humans^34^. This represents a major public health concern, as excessive exposure to metals such as copper (Cu), zinc (Zn), and manganese (Mn) has been linked to increased risks of respiratory and neurological disorders^35–37^.

Dust generated from oilseed processing may contain toxigenic fungi, mycotoxins, and heavy metals that pose risks to human health, particularly respiratory illnesses; this represents one of the key research gaps. However, no studies have been conducted in Ethiopia to assess the respiratory symptoms consequences of dust PM emissions from oil-seed processing facilities. Consequently, conducting occupational health and safety research in oilseed processing factories is essential to assess and address these risks.

To our information no previous studies assessed oil seeds dust PM exposure and respiratory symptoms for OSP factories. The respiratory health of working in OSP factories of Ethiopia has not yet been explored. It is projected that about 15 million people in Ethiopia rely directly or circuitously on oil manufacture for their livings^13^. However, no study has yet been conducted on oil-seed dust exposure and its associated respiratory symptoms and contributing factors, highlighting the novelty and current importance of the present study. Therefore, facts gathered by this study will add more knowledge about respiratory health and seeds dust exposure and this can aid health developers and other pertinent sponsors to implement preventive strategies.

No methodical soundtrack of occupational respiratory diseases in OSP in Ethiopia; So that will help minimize illness, improving work place health &endorsing best practice. The study’s findings also provide baseline information for related studies. This study will help for evidence-based interference, rule attention and likely investigate direction in the upcoming. Future interventions study can be benefited from this study, like health education for health and safety practice enhancement in reducing occupational diseases exposure. The findings of this study will be invaluable to policymakers and the Ethiopian government in assessing existing conditions related to dust exposure, ventilation systems, and the use of personal protective equipment (PPE) across all factories, including oil seed processing (OSP) industries.

This study provides evidence to support governmental efforts to strengthen workplace safety strategies, public health prevention measures, and the promotion of safer working environments for manufacturing workers. Accordingly, the study aimed to determine particulate matter exposure levels and identify factors associated with respiratory symptoms among oil-seed processing factories workers.

## Methodology

### Study area, location, design and period

A factory-based cross-sectional study was conducted in Adama City, one of the largest urban centers in Ethiopia after Addis Ababa. Adama is located in eastern Ethiopia within the Oromia Regional State at approximately 8°33′ N latitude and 39°16′ E longitude, about 90–100 km southeast of Addis Ababa by road, and covers an estimated area of about 130 km^2^^38,39^. Baseline data were collected from January 1 to February 2, 2025, followed by a second round of data collection from August 18 to October 18, 2025, to assess seasonal variation in workplace particulate matter (PM) exposure.

### Population

The source population consisted of all OSP factories and all OSP factory workers in the Oromia Regional State. The study population included all OSP factories located in Adama town and the East Shewa Zone, as well as all workers employed in these factories. The study participants were drawn from OSP factories in Adama town selected through simple random sampling, along with their workers who were employed during the study period. Eligible participants were workers aged 18 years and above who were working in the selected OSP factories in Adama town, Oromia Region, at the time of the study.

#### For inclusion and exclusion criteria: the following inclusion criteria were applied

Suitable participants included workers aged ≥ 18 years who had been employed for at least one year in operational oil-seed processing factories in Adama City, Oromia Regional State, during the study period.

**Exclusion criteria: The following exclusion criteria were applied**

Factories operating for less than one year during the study period and workers aged ≥ 18 years with less than one year of employment were excluded. Administrative staff were also excluded from the study.

**Operational definition**

- **Occupied unit:** Is the working environment specific place where worker is doing activity and space is occupied by this worker during working time.

### Sample size estimation

To estimate the prevalence of respiratory symptoms among workers in Ethiopian oil seed processing (OSP) factories, the single population proportion formula was used. Since no previous studies had been conducted in OSP factories, a prevalence of 50% was assumed.

${\boldsymbol{n}}=\frac{{\left(\frac{{\boldsymbol{Z}}{\boldsymbol{a}}}{2}\right)}^{2})\mathbf{*}\mathbf{p}\left(1-{\boldsymbol{p}}\right)}{{{\boldsymbol{d}}}^{2}}$ Wherever n; desired illustration extent; Z; Z figure for a near of sureness 95% (Zα/2 = 1.96).

P = predictable pervasiveness or quantity (in amount of one; if 50%, P = 0.5), Because there is no study done about this among OSP factory workers.

d = exactness (in quantity of one; if 4%, d = 0.04). n = 600.25 → n = 600; So n = 600.25 which mean 600 by including the non-response rate of 20% was taken. It becomes 120.05 + 600 = 720.3. So, total sample size be comes 720.

This assumption was made to account for possible losses due to various unforeseen reasons; however, it was expected that the final loss of participants would be minimal.

Ultimately, the largest calculated sample size was selected. During data collection, a small number of workers did not respond, resulting in a nonresponse rate of 0.55%**,** and the final sample size was 716 participants**.**

Losses occurred when some workers left during the year due to retirement, illness, transfers, job changes, or resignation. Missing participants were addressed by revisiting participants on more convenient dates and by collecting the required information on alternative working days.

Selection bias was minimized through the use of simple random sampling. A total of 14 factories were selected due to time and logistics constraints. According to WHO guidelines, selecting a minimum of 30% of industries is sufficient for research purposes.

### Sampling distribution/procedures

Out of the 33 existing oil seed processing (OSP) factories, 14 were randomly selected. The calculated sample size was then proportionally allocated to each selected factory based on the number of workers. A sampling frame was established, and the sample size was proportionally allocated to all selected factories. Finally, 716 workers were chosen from these factories using simple random sampling, including all eligible employees except administrative staff**.**

In qualitative study using purposive sampling, a total of 19 participants were selected for the study, including 6 factory workers, 2 health professionals, 4 factory managers, 2 employers, 2 kebele leaders, 1 city administration representative, 1 city health office representative, and 1 community representative. The study involved observation using checklist, 2 focus group discussions (FGDs) and 18 in-depth interviews (IDs).

### Working area particulate matter measurement

One common approach is static (area) measurement**,** where a sampling instrument is placed at a fixed point within the work area to determine dust concentration in that specific location. In this study, the area measurement method was employed to assess dust concentration using Pats + , Daylos DC1900, and Laser PM2.5 Meter-5800D/5800E for at least eight hours per day. Although personal measurement is the preferred approach, this method was not selected because it is costly, individual monitoring devices are not readily available, and wearing the instruments is uncomfortable for workers during work activities. In addition, workers were not willing to carry the devices.

Although workers moved across different sections, static (area) measurement method provided a reliable estimate of overall dust exposure levels. Air quality for PM_10_ and PM_2.5_ was measured in each work section twice a year. The first measurement was conducted during the wet season (August) and the second during the dry season (January) to justification for consistent climate differences^40^.

### Data collection, tools and quality assurance mechanisms

#### Location in working sections

The devices were connected in each main occupied unit at least 1.5 m away since windows and doors, located at a tallness of 1.5 m above the ground. They were also positioned 1.5 m parallel from any initial stages to diminish the entrance of ambient air and located safely to avoid meddling with normal factory processes or turbulences^41^.

#### Data quality

Field workers appropriate full training given on the operation of sampling equipment, counting PATS + , Dylos DC1900, and Laser PM2.5 Meter-5800D/5800E, using a detailed manual with visual guides^42^. To ensure that each 8-h sampling period accurately represented typical working conditions, PM_2.5_ concentration data were excluded from analysis if the total sampling duration was less than eight hours^43^. The PATS + , Dylos DC1900, and Laser PM2.5 Meter-5800D/5800E (detection range: 0–999.9 µg/m^3^) devices across 336 workplaces to verify consistency and accuracy. Prior to and following deployment in each work section, all nursing tools were zero-calibrated by being sealed in a plastic bag for 10 min^40^. The controlling instruments were connected roughly 1.5 m above the ground and within 1.5 m of openings and entrances. Quantities of PM_2.5_ and PM_10_ captivations in the work areas were gotten by means of PATS + , Dylos DC1900, and Laser PM2.5 Meter-5800D/5800E devices, which have a recognition range of 0–999.9 µg/m^3^ and a smallest particle discovery size of 0.3 µm^44^. Each monitor was zero-calibrated by means of a filter both before and after each sampling session^44^. Calibration of the particulate matter (PM) monitoring instruments—PATS + , Dylos DC1900, and Laser PM_2.5_ Meter-5800D/5800E was conducted by comparing their measurements with those of a higher-accuracy reference monitor. Correction factors were applied to account for sensor drift, particle composition, and variations in ambient humidity. In addition, the Dylos device was collocated with a calibrated regulatory-grade reference instrument (e.g., a Federal and Oromia Region Environmental Protection Authority reference method sampler) for an extended period. A zero check (zero calibration) was also performed to confirm that the sensors recorded zero values under clean-air conditions.

Regular cleaning was performed by routinely the fan, laser path, optical chamber, and sampling inlets, as dust accumulation on these components can lead to significant and persistent measurement errors, including falsely elevated readings.

Calibration of the particulate matter (PM) monitoring instrument (PATS +) was performed by comparing its measurements with those of a higher-accuracy reference instrument to account for measurement errors and environmental influences. A stable reference standard—such as a gravimetric sampler or a calibrated, high-quality optical particle counter (e.g., GRIMM or DustTrak) was used to derive appropriate correction factors. The PATS + device was co-located with a regulatory air quality monitoring station (GAMS) for a minimum of 100 h to establish a robust correlation. Data from both the PATS + (test instrument) and the reference monitor were recorded simultaneously, ensuring that calibration covered the full operational range, from low to high particulate matter concentrations.

Previous field validation studies have demonstrated that PATS + , Dylos DC1900, and Laser PM2.5 Meter-5800D/5800E show good agreement with gravimetric methods, which are considered the gold standard for monitoring indoor air pollution^40^.

Moreover, before and after deployment in the work sections, all PATS + , Dylos DC1900, and Laser PM2.5 Meter-5800D/5800E devices were zero-calibrated inside a plastic bag for 10 min. To verify proper functioning and ensure comparability with the reference instrument, additional measurements were conducted outside dusty work areas at locations free from dust sources to assess baseline normality. When the instruments produced comparable readings under these conditions, field measurements were initiated. All devices were operated simultaneously at the same location and time, and their results were compared. As calibration was performed specifically for this study, relative differences among monitors were evaluated prior to actual sampling. Each monitor was zero-calibrated using a filter both before and after every sampling session. The instruments were set to real-time monitoring mode with a 1-min PM_2.5_ measurement interval.

A professional expert in PM measurement instruments accompanied the data collectors throughout the data collection process and closely supervised all measurement procedures. In addition, the data collectors received three days of intensive training on particulate matter measurement techniques and data collection protocols prior to field deployment.

#### Respiratory symptoms health examinations

Respiratory symptoms (yes/no) were evaluated using the consistent form developed by the American Thoracic Society (ATS)^45^. Both interviews and observations were carried out by trained data collectors. A worker symptom recorded if they had experienced cough, phlegm, wheezing, dyspnea, chest pain, chest tightness, or breathlessness lasting for at minimum 3 months within the previous year.

#### Data quality assurance mechanisms

During the design phase of the data collection instruments, all questions and checklists were carefully developed to ensure consistency with the study variables and overall research objectives. The tools were structured to capture all relevant components of each variable and incorporated standardized instruments previously validated in other studies. Calibration and standardization of measurement devices were conducted to enhance validity. The questionnaire underwent peer review during the initial stage to assess its validity and reliability. As it was adapted from standardized tools developed by the World Health Organization (WHO) and the Occupational Safety and Health Administration (OSHA), and had been used in previous studies, it was deemed appropriate for this research. A pretest was conducted outside the study area prior to the actual data collection. To ensure reliability, data collectors received three days of training, instruments were calibrated before use, and experienced professionals were assigned for supervision. Participants of both sexes were included. In addition, two follow-up visits were conducted, and daily on-site supervision was carried out by the principal investigator throughout the data collection process. Regarding external validity, selection bias was minimized through random selection of study factories and by including all eligible workers except administrative staff from each selected factory. Furthermore, the inclusion of both private and government-owned factories ensured representation of all factory types in eastern Ethiopia. Potential sources of bias were controlled through the use of multiple researchers and data collection methods, the inclusion of diverse participants, and blinding of participants, researchers, and data analysts to reduce the influence of subjective information on outcomes. Confounding was minimized by design strategies (randomization, restriction, matching) and statistical adjustments (stratification, regression), ensuring the observed association between exposure and outcome is as close to causal as possible. Confounding was controlled through restriction, stratification, and statistical adjustment during analysis. The findings from the selected oilseed processing factories can be generalized to other OSP factories. Additional strategies included matching (participants by age, sex), randomization (Assigning workers randomly to group A: uses PPE and group B: Does not use PPE; Exposed to PM above WHO standard and un exposed), intention-to-treat analysis to address participant attrition, and complete and transparent reporting of data and study outcomes.

#### Ethics declarations

To conduct the study, permission was obtained from both the industries and their workers. Ethical approval and clearance were secured from the Ethical Review Board of the Jimma University Institute of Health Sciences. The Institutional Review Board of Jimma University gave ethical clearance with the reference number JUIH/IRPB/213/25. Official letters were also sent to the Adama town and East Shewa Zone administrations, who voluntarily supported the study. All participants were informed about the study procedures, and interviews were conducted only after obtaining written informed consent. Participants were assured of confidentiality throughout their involvement in the study.

### Data management and statistical analysis

Quantitative descriptive data were analyzed using STATA version16.12. In this research Microsoft Excel was used to transfer data from the instruments, to process and analyze data exported from the measurement instruments. SPSS was used for template preparation, cross-tabulation, and preliminary data analysis, while STATA was used for advanced data analysis.

Questionnaires and raw environmental measurement data were used to assess exposure risks related to health hazards. Statistical software was employed for data entry and cleaning. Appropriate statistical tools, such as cross-tabulation and frequency analysis, were applied, and missing data were checked in SPSS to ensure the validity and reliability of the study.

Binary and multivariable logistic regression analyses were conducted to identify predictors. Variables with a p-value ≤ 0.02 in the binary logistic analysis were selected as candidates for the multivariable logistic regression, and statistical significance was determined at p-value < 0.05.

Using purposive sampling**,** a total of 19 participants were selected for the study, including 6 factory workers, 2 health professionals, 4 factory managers, 2 employers, 2 kebele leaders, 1 city administration representative, 1 city health office representative, and 1 community representative. The study involved 2 focus group discussions (FGDs) and 18 in-depth interviews (IDs)**.** Verbatim transcriptions and field notes collected from the factories were analyzed using qualitative content analysis with the aid of ATLAS.ti23 software.

Additional qualitative data from follow-up discussions, worksheets, observational checklists, and annotations were also incorporated into the analysis. Disagreements in coding were resolved through iterative review, and data saturation was confirmed when coders identified no new codes from the transcriptions and field notes. Finally, all data analysts and the authors reviewed and verified the codes and their categories. Both quantitative and qualitative results were categorized to identify relationships within the overall research context. Finally presented using tables and figures, displaying the corresponding percentages to reflect participants’ responses to each question.

## Result

### Socio-demographic characteristics

The study included 716 respondents, of whom 357 (49.9%) were female. The mean age of participants was 33.55 ± 7.79 years, with a geometric mean of 32.69 ± 7.79 years. The response rate was 99.44%**.** Among the participants, 365 (51%) were aged 25–35 years, 208 (29.1%) had primary-level education (grades 1–8), and 416 (58.1%) fell into the corresponding category shown in Table 1. Age, income and working hour were categorized rendering to previous study done^46^.

**Table 1:** Socio-demographic characteristics of oil seed processing factory workers (n = 716).

Variables	Frequency	Percent	Variables	Frequency	Percent
Age of respondents(46) 18–24 year 25–35 year above35 year	70 365 281	9.8 51 39.2	Marital status Single Married Widowed Divorced	265 290 54 107	37 40.5 7.5 14.9
Working hour 0-8 h above 8 h	354 362	49.4 50.6	Sex of Respondents Male Female	359 357	50.1 49.9
Income of workers(46) • 0–2000 E.Birr • 2001–2900 E.Birr • 2901-4050E.Birr • above4050E.Birr	53 49 262 352	7.4 6.8 36.6 49.2	Previous history of respiratory diseases No Yes	709 7	99 1
Working service years 0–5 years Greater than 5 years	300 416	41.9 58.1	Participants work shift No Yes	48 66.8	6.7 93.3
Participants job rotation No Yes	49 667	6.8 93.2	Type of employment Temporary Permanent	354 362	49.4 50.6
Educational status primary1-8 grade secondary9-12 grade technical or diploma Bsc degree &above	208 160 145 203	29.1 22.3 20.3 28.4	Participants job satisfaction No Yes	526 190	73.5 26.5
Previous dust exposure history in the last 5 years No Yes	663 53	92.6 7.4	Family history of chronic respiratory diseases No Yes	714 2	99.7 0.3

### Occurrence of work-related respiratory indicators

Disease pervasiveness throughout study, 181(25.3%) respiratory symptoms, 181(25.3%) were bronchitis symptoms, 166(23.2%) described wheezing problems, 161(22.5) phlegm, 160 (22.3%) Dyspnea, 156(21.8%) sneezing problems,120(16.8%) chest illness,107(14.9%) cough and 90 (12.6%) were recounted workers faced occupational asthma symptoms in the last3 months.

The arithmetic mean (AM ± SD) and geometric mean (GM ± SD) concentrations of PM₂.₅ exposure among OSP factory workers were 19.72 ± 11.8 µg/m^3^ and 15.75 ± 11.8 µg/m^3^, respectively. Similarly, the AM ± SD and GM ± SD concentrations of PM₁₀ exposure were 55.24 ± 14.5 µg/m^3^ and 53.40 ± 14.5 µg/m^3^, respectively (Table2).

**Table 2:** PM _2.5_ and PM_10_ concentrations in oil seed processing factories.

List of factories	Parameter measurement for two seasons					
	Mean of PM_10_ (µg/m^3^)		Mean of PM _2.5_(µg/m^3^)		Mean of PM_10_ (µg/m^3^)	Mean of PM _2.5_ (µg/m^3^)
	Summer AM(GM)	Winter AM(GM)	Summer AM(GM)	Winter AM(GM)	Cumulative of two season AM(GM)	Cumulative of two seasons AM(GM)
Factory 1	51.93(50.01)	43.54(42.84)	16.94(14.92)	10.58(9.49)	47.73(46.42)	13.76(12.2)
Factory 2	62.82(59.6)	55.74(53.56)	22.45(19.54)	20.35(16.84)	59.28(56.58)	21.40(18.19)
Factory 3	56.7(54.44)	52.95(51.17)	19.01(15.89)	16.45(12.5)	54.82(52.8)	17.73(14.19)
Factory 4	58.97(57.1)	56.18(54.65)	19.47(15.57)	17.64(13.05)	57.57(55.87)	18.55(14.31)
Factory 5	54.77(54.75)	54.13(52.65)	21.01(18.16)	18.89(15.14)	54.45(53.7)	19.95(16.65)
Factory 6	54.77(53.26)	53.45(51.9)	18.43(14.3)	16.53(11.55)	54.11(52.58)	17.48(12.92)
Factory 7	61.42(60)	60.06(58.62)	25.51(22.21)	14.02(20.2)	60.74(59.31)	19.76(21.2)
Factory 8	57.44(56.23)	56(54.71)	23.7(20.52)	22.06(18.41)	56.72(55.47)	22.88(19.46)
Factory 9	63.91(62.92)	62.55(61.5)	28.59(25.45)	27.24(23.76)	63.23(62.21)	27.91(24.60)
Factory 10	56.51(54.72)	55.34(53.57)	19.15(14.91)	17.44(12.62)	55.92(54.14)	18.29(13.76)
Factory 11	58.49(57.4)	55.74(54.28)	22.38(18.97)	21.05(17.15)	57.11(55.84)	21.72(18.06)
Factory 12	56.29(54.76)	53.85(52.13)	19.1(15.11)	17.1(12.52)	55.07(53.44)	18.1(13.81)
Factory 13	51.67(49.19)	49.94(47.61)	15.83(11.83)	13.76(9.28)	50.81(48.4)	14.79(10.56)
Factory14	47.94(45.91)	45.38(43.76)	16.06(13.31)	14.26(10.9)	46.66(44.83)	15.16(12.10)
Average	56.68(54.69)	53.80(51.99)	20.84(17.05)	18.60(14.22)	55.24(53.40)	19.72(15.75)

PM_2.5_^_^ Particulate matter with diameter of 2.5 µm (µm) or less, PM_10_^_^ Particulate matter with diameter of 10 µm (µm) or less, AM- arithmetic mean, GM-geometric mean.

The comparison of PM according to WHO category with respiratory symptoms was seen and it was higher for above standards (Table3).

**Table 3:** Comparison of PM_2.5_ and PM_10_ exposure with respiratory symptoms among oil seed processing factory workers.

Symptoms	PM _2.5_ Concentration				PM _10_ Concentration			
	0–15 (µg/m^3^)		> 15 (µg/m^3^)		0–45 (µg/m^3^)		> 45 (µg/m^3^)	
	Num	%	Num	%	Num	%	Num	%
Dyspnea	31	4.33	129	18.02	43	6	117	16.3
Chest pain/illness	28	3.91	92	12.85	40	5.6	80	11.2
sneezing	27	3.77	129	18.02	43	6	113	15.8
Cough	46	6.42	61	8.52	40	5.6	67	9.4
Wheezing	34	4.75	132	18.43	49	6.8	117	16.3
phlegm	30	4.19	131	18.3	47	6.6	114	15.4
Bronchitis symptoms	36	5.03	145	20.25	53	7.4	128	17.9
Shortness of breath	24	3.35	119	16.62	40	5.6	103	14.4
Respiratory symptoms	36	5.03	145	20.25	53	7.4	128	17.9
Asthma	20	2.79	70	9.78	22	3.1	68	9.5
Total^@^								

^@^Total is not applicable because of more than one case for one participant. PM_2.5_^_^ Particulate matter with diameter of 2.5 µm (µm) or less, PM_10_^_^ Particulate matter with diameter of 10 µm (µm) or less.

The WHO 24-h air quality guideline limits are 15 µg/m^3^ for PM₂.₅ and 45 µg/m^3^ for PM₁₀, while the annual guideline limits are 5 µg/m^3^ for PM₂.₅ and 15 µg/m^3^ for PM₁₀^47^. The European Union, which applies air quality standards similar to those used in Germany, sets a 24-h limit of 25 µg/m^3^ and an annual mean limit of 10 µg/m^3^ for PM₂.₅, an annual mean limit of 20 µg/m^3^ for PM₁₀, and a 24-h mean limit of 45 µg/m^3^ for PM₁₀^48^.According to the U.S. Environmental Protection Agency (EPA) National Ambient Air Quality Standards, the primary annual average limit for PM₂.₅ is 9 µg/m^3^, not to exceed 15 µg/m^3^, calculated as a three-year average and revised to enhance health protection. The 24-h average limit for PM₂.₅, applicable to both primary and secondary standards, is 35 µg/m^3^, based on a three-year average. For PM₁₀, the 24-h average limit for the primary standard is 35 µg/m^3^, while the secondary standard is set at 150 µg/m^3^^49^.

### Binary logistic regression and multivariable logistic regression analysis for PM concentration and respiratory symptom

Eleven variables with a p-value ≤ 0.02 were selected as candidates for the multivariable logistic regression analysis. The results showed that workers exposed to PM₂.₅ concentrations > 15 µg/m^3^ were 18.6 times more likely to experience respiratory symptoms compared with those exposed to ≤ 15 µg/m^3^ (AOR = 18.6, 95% CI: 9.12–37.43). Workers who did not use personal protective equipment (PPE) were 18.3 times more likely to develop respiratory symptoms than PPE users (AOR = 18.3, 95% CI: 2.39–39.39). Additionally, participants who used wood as a household energy source were 15.47 times more likely to report respiratory symptoms than those who did not (AOR = 15.47, 95% CI: 4.25–36.40) (Table 4).

**Table 4:** Binary and multivariable logistic regression analysis of factors associated with dust (PM) exposure and respiratory symptoms (n = 716).

Variables	Respiratory symptoms		Odds Ratio (95% C.I.)			
	No	Yes	COR (95%CI)	p-value	AOR (95%CI)	p-value
	Number	Number				
Gender of respondents Male Female	194 341	165 16	1 18.13(10.54–31.19)	1 0.000	1 0.03(0.005–1.193)	0.056
Type of employment Temporary Permanent	239 296	115 66	1 0.46(0.33–0.66)	0.000	1 0.065(0.013–1.32)	0.081
Working hour 0-8 h Greater than 8 h	241 296	113 68	0.49(0.35–0.69) 1	0.000	0.029(0.004–1.203) 1	0.080
Work place dust PM_2.5_ concentration • ≤ 15 µg/m^3^ • > 15 µg/m^3^	313 222	36 145	1 5.68(3.80–8.50)	0.000	1 18.6(9.12–37.43)	0.022*
Type of energy used at home • Electric city • Kerosene • Wood • charcoal	195 128 105 107	30 38 62 51	1 1.93(1.14–3.27) 3.84(2.34–6.30) 3.1(1.90–5.15)	0.015 0.000 0.000	1 3.71(1.89–15.51) 15.47(4.25–36.40) 11.90(3.03–26.62)	0.072 0.000* 0.000*
Ventilation of work place Poor Good	146 389	164 17	25.7(15.06–43.86) 1	0.000 1	14.29(3.01–26.70) 1	0.001*
There is work place Supervision No Yes	341 196	65 116	3.14(2.21–4.46) 1	0.000	0.5(0.08–3.11) 1	0.459
PPE use Poor good	345 190	65 116	3.24(2.30–4.61) 1	0.000	18.3(2.39–39.39) 1	0.005*
There was training about OHS No Yes	342 193	15 166	19.6(11.23–34.23) 1	0.000	3.01(2.01–4.02) 1	0.000*
Alcohol consumption No Yes	440 95	118 63	1 2.47(1.69–3.61)	1 0.000	1 1.37(0.16–11.74)	0.774
Smoking cigarettes No Yes	458 77	126 55	1 2.59(1.74–3.86)	0.000	1 0.98(0.10–9.63)	0.990

NSI = Not significantly associated because p > 0.05 or touch 1, *significantly associated, COR-crude odds ratio, AOR-adjusted odds ratio.

**Fig. 1:**
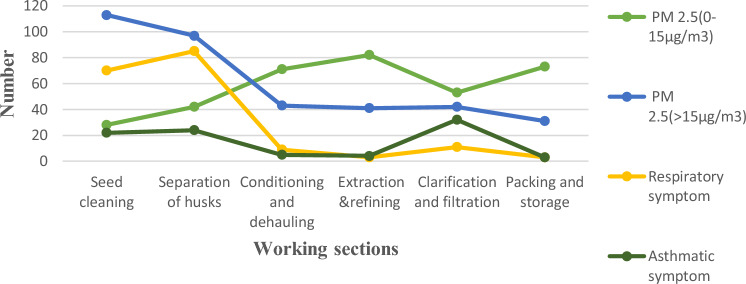
PM2.5 concentration versus respiratory and asthma symptoms in working sections. PM_2..5_^_^ Particulate matter with diameter of 2.5 µm (µm) or less, PM_10_^_^ Particulate matter with diameter of 10 µm (µm) or less.

**Fig. 2:**
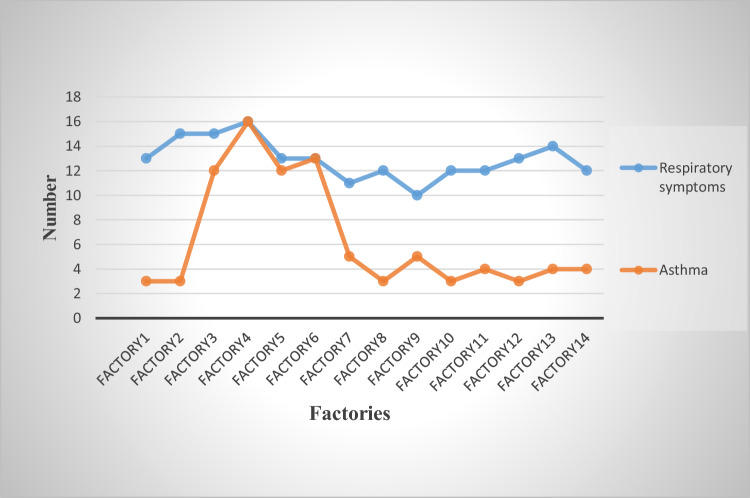
Respiratory and asthma symptoms in factories.

**Fig. 3:**
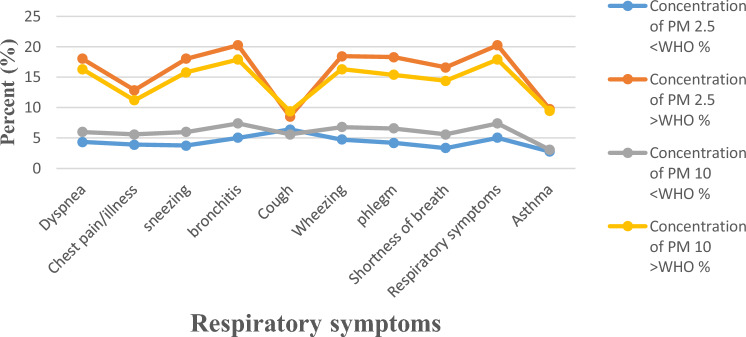
PM2.5 and PM10 concentration inrelation to prevalence of respiratory symptoms. PM_2.5_^_^ Particulate matter with diameter of 2.5 µm (µm) or less, PM_10_^_^ Particulate matter with diameter of 10 µm (µm) or less.

The particulate matter (PM) concentrations in relation to respiratory and asthma symptoms, by working sections and factory level, as well as the WHO PM standards related to respiratory symptoms, are presented in Figs. 1, 2, and 3 below.

## Qualitative study findings

Factories of OSP were categorized into three major themes with seven related subthemes: knowledge gaps; training; shortage of PPE; PM exposure; risk assessment; ventilation and supervision. This component of the study employed a qualitative design (n = 19). Data were collected primarily through semi-structured and unstructured questionnaire 18 in-depth interviews, as well as 2focus group discussions (FGDs).

Limited knowledge of occupational health and safety (OHS) among workers in oil seed processing (OSP) factories has been identified as a key barrier to maintaining and improving their health status. A43-years-old man response ability: *“I don’t have knowledge regarding dust exposure and safety I heard from my friends but not as much in detail of course including respiratory health…”* (FGD 2, A 43 old man, informant-16). A male worker shared similar spirits: *“No any one knows about occupational health and safety in brief there is lack of knowledge except its little dust exposure information…”* (A male worker, in-depth interview six, informant-17).

No occupational health and safety training has been provided to worker. A46-years-old man response an idea*: “No one decided and gave training for workers until now, so that it is mandatory to give training about risk and safety…unless nothing can easily improve…”* (A male worker, in-depth interview twelve, informant-1). A24-years-old woman response similar idea: *“Education or training is one of most important tools that makes workers alert about safety and respiratory risk control. I did not take any training about this; hopeful I will get this in future…”* (FGD 2, A 24 old women, informant-10).

Supportive supervision related to occupational health and safety and exposure to particulate matter is also inadequate among factory workers. A39-years-old man response ability: *“…one of the gaps is I think lack of supportive supervision regarding occupational health and safety that can make us not to improve our health and safety… management has to decide…”* (A male worker, in-depth interview five, informant-11). A female worker shared similar spirits: *“Supervision is needed for our safety at work and if there is supervision everything can be improved…in this factory…”* (FGD 1, A 49 old women, informant-5).

Participants also reported the absence of orderly workplace risk assessment. A44-years-old woman response idea: *“The identification of risk regularly can facilitate occupational health and safety application at work place.”* (FGD 2, A 44 old woman, informant-2). A 24-years-old women response similar idea: *“Assessing of hazards can easily identify and facilitate reduction of dust…and safety in the work place of workers.”* (FGD 1, A 24 old woman, informant-4).

This qualitative study identified inadequate workplace ventilation as a significant gap. A31-years-old woman response ability: *“…Ohhh…anybody can guess…. that one of the gaps is there is no any ventilation installed in dusty areas…risk of workers to diseases like respiratory…”* (A female worker, in-depth interview nine, informant-13). A28-years-old man response skill: *“…one of the gaps is I think lack of aeration system around working areas…. can hide from dust exposure regarding health and safety that can make us not to improve our respiratory health and safety…”* (A male worker, in-depth interview seven, informant -12).

Participants highlighted that insufficient availability of personal protective equipment (PPE) remains a major workplace challenge. A 45-year-old male worker reflected: *“…Of course, it is not easy to work safely without PPE. The shortage of PPE is one of the factors contributing to the respiratory health status of workers…”* (Male worker, in-depth interview 8, Informant-14). A 41-year-old woman expressed a similar view: *“…It is difficult to obtain PPE easily in this factory. Therefore, if workers’ health and safety are to be ensured, this problem needs to be addressed…”* (FGD1, 41-year-old female participant, Informant-6).

Study participants indicated that workers are at momentous risk of particulate matter exposure. A 27-year-old woman expressed a similar view: *“….in terms of dirty dust in the work place no one consider it….my friend very ill due to respiratory illness when she is handling husk removing for certain months…”* (A female worker, in-depth interview ten, informant-8).

Qualitative content analysis was conducted after data collection, following the procedures outlined in Tables 5 and 6.

**Table 5:** Phases of content analysis and theme development in the qualitative data.

SN	Phases	Explanation
1	Transcription of data	Audio-recorded interviews, supplemented by handwritten field notes, were transcribed verbatim for analysis
2	Familiarization with data	Familiarization with the interview data was achieved by repeatedly listening to the audio recordings and reviewing the transcripts alongside contextual and reflective field notes
3	Generating initial codes	The transcripts were carefully examined line by line, and meaningful segments were paraphrased or assigned descriptive codes to capture key ideas identified in the text
4	Searching for themes	The authors reviewed and organized the codes based on similarities in content, which facilitated the identification of potential themes. Subsequently, theme names were developed to comprehensively represent the grouped codes within each theme
5	Reviewing themes	The identified themes were reviewed against the coded data extracts and evaluated to ensure they accurately represented the overall dataset
6	Defining and naming themes	The principal investigators refined the names and definitions of each theme and developed concise descriptions to clearly characterize them
7	Producing report	The authors prepared a comprehensive analytical report using the most relevant data extracts and linking the findings to the original research question. The report was subsequently reviewed and approved by all authors

**Table 6:** Themes, subthemes, and formulated meanings derived from interviews with workers.

No	Theme description	Theme components	Thematic summary
1	…I don’t have knowledge regarding dust exposure and safety I heard from my friends but not as much in detail of course…”	knowledge regarding dust exposure	Poor Knowledge
2	…attributed their reluctance to discussing occupational risk with service users to lack of formal training	Risk exposure Lack of training about risk	Lack of training
3	In terms of dirty dust in the work place no one consider it….my friend very ill due to respiratory illness when she is handling husk removing for certain months	Dust particles exposure	PM exposure
4	…evidence we have is that it is worth giving most people aware off the danger in order to see if their illness would be a cause of work or not…follow up is mandatory…	Awareness Needs supervision	No supervision
5	“…one of the gaps is I think lack of aeration system around working areas…. can hide from dust exposure regarding health and safety that can make us not to improve our health and safety…	Aeration system in work- place	In adequate Ventilation
6	“…Of course, it is not easy to work safely without PPE. The shortage of PPE is one of the factors contributing to the poor health status of workers…	Shortage of PPE is challenge	Shortage of PPE
7	…do my duties during the whole work shift without donning the appropriate safety equipment. I will use PPE without restrictions if I can access it. However, the industry has neither a strategy nor motive to offer the proper protective clothing	Access of PPE is lacking	Lack of PPE
8	The identification of risk regularly can facilitate occupational health and safety application at work place…	Regular Risk assessment	No risk assessment
9	…don’t always use mask but sometimes this due to my coverall is tear and hole on clothes, for continuous use of masks, it is also dirty and not clean enough to work properly…work cloth is not washed regularly in this work place…	Not use PPE Tear/worn out gowns /work cloth/uniform, Not washed	Not use of clean PPE

### Observation using checklist

Results from workplace observations in OSP factories indicated several deficiencies in dust control and workplace safety practices. Inadequate technical measures were used to regulate PM dust exposure, and poor indoor air quality was evident across many sections of the factories. Although entryways and windows were present, effective airflow was limited in several work areas due to poorly organized workspaces and suboptimal layout of machinery and workstations.

Most employees were not provided with appropriate PPE. Among the few workers who received PPE, proper and consistent use was uncommon; some handled it carelessly or left it unattended at their workstations. Additionally, workers generally did not wear standard uniforms or gowns. Even when uniforms were used, they were often torn, dirty, and poorly maintained.

Although some PPE was available, it was frequently damaged, poorly managed, and sometimes taken home by workers due to the absence of adequate administrative control and monitoring systems. Overall, these observations suggest that the unfavorable workplace environment may partly explain the high prevalence of respiratory symptoms reported in this study.

## Discussion

This study measured dust particulate matter exposure and assessed respiratory health risks among workers in oil seed processing (OSP) factories.

Unlike previous studies, which often assessed respiratory symptoms without directly measuring particulate matter, relying instead on questionnaires that asked participants whether dust was present in the factories and then concluded respiratory symptoms to dust exposure. Additionally, previous studies were typically conducted in only one or two factories, and even when multiple factories were included, they often represented mixed industries (e.g., textile, flour, cotton, metal, cement, or others). Finally, these studies generalized their findings to all industries. Though, factories differ in terms of the raw materials used, processing mechanisms, seasonal variations in particulate matter concentrations, workforce characteristics, and exposure levels.

However, this study was conducted exclusively in oil-seed processing (OSP) factories using a random sampling method. Similarly, participants were also selected by simple random sampling methods. Several studies have reported fungal contamination in various oil-bearing seeds, as well as the presence of mycotoxins in the oils-seeds and extracted oil from these seeds^28,29^. For this reason, investigating particulate matter exposure and its association with respiratory symptoms is considered essential. Particulate matter concentrations were measured concurrently with the assessment of respiratory symptoms, providing a more specific analysis.

Qualitative findings supported the quantitative results and were integrated to enhance the discussion. The prevalence of respiratory symptoms among OSP workers was 25.28%, which is slightly higher than that reported in other studies conducted in Ethiopia (21.7%) but not OSP. This difference may be attributed to variations in study design, population characteristics, exposure levels, and types of processing factories.

Similar results have been reported in studies from Tanzania, Ethiopia, Papua New Guinea, and Uganda^50–52^. These similarities may be attributed to comparable working environments, awareness of workers, OHS information they have, and the prevention and control measures in place.

In this study, 12.57% of workers reported asthma. In contrast, another study reported that 53.2% of participants experienced at least one respiratory symptom prior to the study period^53^. Compared with the present study, that study reported higher prevalences of sneezing (62.2%), cough (45.9%), chest tightness (37.2%), shortness of breath (36.5%), runny or blocked nose (31.1%), wheezing (8.8%), and asthma (1.4%)^53^.

In this study, the prevalence of most respiratory symptoms was lower than that reported in other studies; however, the prevalence of bronchitis symptoms, wheezing, and asthma was comparatively higher. These variations and inconsistencies may be explained by differences in the implementation of prevention and control measures, levels of worker awareness, types of raw materials used, factory characteristics, PM concentration and access to occupational health and safety information.

Finding of pooled prevalence of respiratory symptoms among industrial workers was 51.6%^54^. In comparison, the prevalence observed in the present study was lower than this pooled estimate. This difference may be attributed to the use of averaged results from multiple studies in the pooled analysis, which can mask variations across individual study settings, as well as the lack of direct particulate matter (PM) measurements in those studies.

In another study, the reported prevalence among participants was 8.1% for cough, 11.7% for phlegm, 6.8% for bronchitis, 0.5% for chronic bronchitis, and 5.5% for chest pain^55^. These rates were lower than those observed in the present study, possibly due to differences in factory type and processing methods, raw materials used, PPE utilization, and the effectiveness of workplace prevention measures.

In statistics, the arithmetic mean (AM) represents the simple average, whereas the geometric mean (GM) better reflects the central tendency of skewed data; therefore, both are commonly reported in air pollution studies. This is the primary reason we used both measures. The AM and GM of PM₂.₅ exposure among OSP workshop workers were 19.72 ± 11.8 µg/m^3^ and 15.75 ± 11.8 µg/m^3^, respectively. For PM₁₀, the AM and GM exposure levels among OSP plant workers were 55.24 ± 14.5 µg/m^3^ and 53.40 ± 14.5 µg/m^3^, respectively. While particulate matter concentrations showed seasonal variation, these differences were not statistically significant across the factories.

The 24-h PM₂.₅ exposure level was below the limits set by the European Union and the U.S. Environmental Protection Agency (EPA)^48^, but exceeded the World Health Organization (WHO) guideline^47^. However, the annual mean PM₂.₅ concentration exceeded the limits of all referenced standards. Similarly, the 24-h PM₁₀ exposure exceeded the standards set by the WHO, the European Union, and the U.S. EPA^49^, and the annual mean PM₁₀ concentration exceeded the limits of all referenced standards. In this study, WHO standards were used as the reference for comparison.

PM₂.₅ and PM₁₀ levels among OSP factory workers exceeded the WHO air quality guideline limits^47^. The WHO 24-h limits are 15 µg/m^3^ for PM₂.₅ and 45 µg/m^3^ for PM₁₀, while the annual guideline limits are 5 µg/m^3^ for PM₂.₅ and 15 µg/m^3^ for PM₁₀^47^.

Moreover, 51.36% of factory workers were exposed to PM₂.₅ concentrations above the WHO guideline limit of 15 µg/m^3^, and 64.53% were exposed to PM₁₀ levels exceeding the WHO limit of 45 µg/m^3^. Consistent with previous evidence, these findings indicate that a substantial proportion of factory workers experience respiratory symptoms^56^.

Among workers exposed to PM₂.₅ levels below the WHO guideline, 48.64% experienced lower exposure, and only 5.03% developed respiratory symptoms. In contrast, among those exposed to PM₂.₅ levels above the WHO limit (51.36%), only 20.25% developed respiratory symptoms.

Similarly, among workers exposed to PM_10_ levels below the WHO guideline (35.47%), only 7.4% reported respiratory symptoms, whereas among those exposed above the WHO limit (64.53%), 17.9% developed respiratory symptoms. These findings clearly demonstrate that exposure to seed-related particulate matter increases the risk of respiratory symptoms.

In the working sections, the highest proportions of workers exposed to PM₂.₅ levels above the WHO guideline were observed in seed cleaning (15.8%), husk separation (13.5%), and conditioning and dehulling (6%), in descending order. The lowest exposure was in the packing and storage section, where only 4.3% of workers were exposed.

Similarly, exposure to PM_10_ above the WHO guideline was most common in seed cleaning (16.1%), separation of husks (12.8%), and conditioning and dehulling (9.5%), while the packing and storage section had the lowest proportion of exposed workers (7.8%).

Respiratory symptoms were most common in the separation of husk Sect. (11.9%), followed by seed cleaning (9.8%), and least common in the clarification and filtration Sect. (1.5%). The prevalence of symptoms decreased from separation of husks to seed cleaning followed by packing and storage, corresponding with declining PM_2.5_ above WHO standard (15 µg/m^3^) (Fig. 1). PM₂.₅ and PM₁₀ concentrations decreased from seed cleaning to packing and storage, making seed cleaning the highest-risk section and packing and storage relatively safer (Figs. 1). Correspondingly, PM₂.₅ and PM₁₀ levels, as well as the prevalence of respiratory symptoms and asthma, showed a steady decline from seed cleaning through packing and storage (Fig. 1).

Respiratory symptoms and asthma increased from Factory 1 to Factories 3 and 4, then declined toward Factories 9 and 10. After Factory 9, both outcomes rose again, reaching higher levels at factories 13 and 14 (Figs. 2). Respiratory symptoms were substantially higher in areas where PM₁₀ and PM₂.₅ concentrations exceeded WHO standards and relatively lower in areas where levels were below the WHO limits (Fig. 3).

The quantitative analysis identified several factors significantly associated with dust exposure and respiratory symptoms among oil-seed processing workshop workers, including high PM₂.₅ levels, use of personal protective equipment, household energy sources, workplace ventilation, and occupational health and safety training. The qualitative findings corroborated these results, highlighting additional workplace challenges such as particulate dust exposure, inadequate supervision and risk assessment, inconsistent use of personal protective equipment, poor ventilation, and limited worker knowledge and training as key contributors to respiratory symptoms.

Lack of training was a significant predictor of respiratory symptoms, consistent with findings from other studies^10,57^. This similarity may be attributed to the substantial influence of behavioral change mechanisms, which are widely recognized across occupational health research.

Other studies support the findings of this study, demonstrating an association between dust particulate matter exposure among employees and respiratory health outcomes^54,58^. This similarity may be due to PM concentrations exceeding WHO guidelines, which are known to cause respiratory symptoms.

Consistent evidence demonstrates that reliance on polluting fuels for household cooking or heating is associated with chronic respiratory diseases^13,14^. This similarity may be attributed to high levels of dust particulate matter exposure exceeding WHO standard limits, inadequate use of personal protective equipment, and poor occupational health and safety practices in factories.

Other studies support these findings, showing that inadequate workplace ventilation significantly increases the likelihood of respiratory symptoms among flour mill workers^59^. Similar results may be attributed to comparable implementation of occupational health and safety systems in both settings.

In this study, non-use of personal protective equipment (PPE) was significantly associated with the occurrence of respiratory symptoms, consistent with findings from previous research^43^. This agreement may reflect the essential role of PPE as an administrative control measure in occupational environments.

The results of this study have implications for future particulate matter (PM) control and reduction, and for the development of new preventive strategies to reduce respiratory symptoms, particularly in oil seed processing (OSP) factories, by policymakers and other concerned bodies.

### The strength of this study

The direct measurement of particulate matter (PM) using instruments, rather than relying solely on questionnaires, allowed for more accurate identification of multiple exposure sources associated with respiratory symptoms. Both factories and study subjects were selected by simple random sampling methods from eligible source. This approach also facilitated differentiation between workers who experienced respiratory symptoms and those who did not. It was supported by qualitative study. Additionally, conducting face-to-face meetings during data collection helped minimize nonresponse bias, clarified questionnaire items, and ensured participation across different socio-demographic groups. Both quantitative and qualitative study was done at the same time. To the best of our knowledge, this is the first study to simultaneously measure dust exposure and respiratory symptoms among workers in oil seed processing (OSP) factories.

### The limitations of this study

Measurement variability may have occurred because workers often moved between different work sections, which could have affected exposure levels. Area sampling used instead of personal sampling or personal exposure measurements were not conducted, which may have increased recall bias and measurement error; Possible instrument error or environmental variation; Therefore, future studies should address this gap. The exposure assessment protocol followed standardized procedures for measuring workplace particulate matter using equipment. However, the measurements were limited to selected sampling periods and locations, which may not fully represent long-term personal exposure levels of workers. Exposure measurements are ideally conducted four to six times per year for each individual; however, in this study measurements were performed only during the dry and wet seasons, which may not fully represent workers’ personal exposure levels. This due to shortage of budget and financial deficiency. Another limitation is the scarcity of literature examining particulate matter (PM) exposure in oil seed processing (OSP) factories. Additionally, the study was conducted exclusively in OSP factories, limiting the generalizability of the findings to other industrial sectors.

A case–control study involving both exposed and unexposed groups, with cohort follow-up, would be more appropriate for establishing causal relationships. Furthermore, long-term follow-up of both exposed and unexposed groups, including pulmonary function assessment, was not included in this study.

## Conclusion and recommendation

Exposure to PM₂.₅ and PM₁₀ from oil seeds among OSP factory workers exceeded WHO guideline limits, with most workers operating under concentrations above these thresholds. The results provide clear evidence that exposure to oil-seeds particulate matter is associated with increased respiratory symptoms. Workers exposed to PM levels above WHO limits experienced higher rates of respiratory symptoms, including asthma, wheezing, phlegm, dyspnea, sneezing, shortness of breath, chest pain, and cough. Factors associated with these symptoms included PM₂.₅ exposure, lack of PPE use, type of home energy source, inadequate ventilation, and insufficient training. Appropriate measures are required to reduce particulate matter exposure, including comprehensive occupational health and safety programs that prioritize source-level dust PM control, regular supervision, engineering controls (such as adequate ventilation), administrative measures (including training and risk assessment), and proper use of PPE. Future studies employing personal exposure measurements and case–control designs are recommended.

### Recommendations

- **For oil seed processing factory management: **
  - Regular PM risk assessments should be conducted to safeguard workers’ health and safety.
  - Continuous awareness programs should be implemented to educate workers on reducing PM exposure and its associated hazards in oil seed processing facilities.
  - Timely and relevant information should be provided to workers to support the management of occupational and environmental health hazards within factory settings.
  - Routine workplace supervision should be enforced to ensure compliance with health and safety practices.
  - Workers should consistently use appropriate PPE to minimize exposure to potential workplace health hazards.

- **For policy makers: **
  - We recommend the effective implementation of comprehensive occupational health and safety programs that prioritize dust reduction at the source, regular workplace supervision, engineering controls (such as adequate ventilation systems), administrative measures (including health education, training programs, and risk assessments), and the provision of appropriate PPE.
  - Furthermore, integrating workplace health and safety programs into broader public health policies and strategies is essential for effectively reducing the burden of occupational hazards in factory settings.

- **For researcher’s: **
  - Future studies should focus on personal PM measurements, assess workers’ knowledge and safety practices related to the prevention of respiratory diseases, and identify specific risk factors. Case–control studies incorporating pulmonary function assessments in relation to dust exposure and respiratory outcomes are recommended, along with investigations into workers’ perceptions of and adherence to PPE use.

## Data Availability

Data are available upon reasonable request. Data will be made available from the corresponding author upon reasonable request following publication with the following below address.
